# Management of Facial Telangiectasias with Hand Cautery

**Published:** 2015-07

**Authors:** Ioannis E. Liapakis, Miriam Englander, Roven Sinani, Eleftherios I. Paschalis

**Affiliations:** 1 Opsis Clinical, Plastic and Reconstructive Surgery, 48 Anogion St., 71304, Therissos, Heraklion-Crete, Greece;; 2 Ophthalmic Consultants of Boston, 50 Staniford St, 02114, Boston, MA, USA;; 3 Department of ENT, ‘’Mother Tereza’’ Hospital of Tirana, SH54 372 Tirana, Albania;; 4 Harvard University, Massachusetts Eye and Ear infirmary, 243 Charles St. 02114, Boston, MA, USA

**Keywords:** Facial telangiectasias, Laser, Electrosurgery, Sclerotherapy, Hand cautery

## Abstract

**BACKGROUND:**

Facial telangiectasias are superficial cutaneous vessels that can result in noticeable aesthetical imperfections. This study presents a technique for the removal of facial telangiectasias using hand cautery.

**METHODS:**

Twenty-five patients with facial telangiectasias were treated using hand cautery (Medicell Inc, Athens, Greece) during 2009-2013. Photo documentation was performed for each patient before and immediately after treatment. Treatment was performed by cauterization at 800°C, delivered via a 30G tip directly to the lesions for milliseconds.

**RESULTS:**

Twenty two out of 25 patients (88%) exhibited complete resolution of telangiectasias using hand cautery. In 5 (20%) patients, single application achieved complete resolution of lesions and in 10 patients (40%) re-treatment was required after 3 weeks. Four patients (16%) required 3 consecutive treatments from which 2 patients (8%) showed slight improvement and one patient (4%) no improvement. No major complications were associated with this procedure except the formation of a white scar in two patients that became inconspicuous after 3 months. Minor complications included skin irritation and edema immediately after the treatment, which resolved within 2-3 days without intervention.

**CONCLUSION:**

Hand cautery is a very safe, effective and inexpensive tool for the treatment of facial telangiectasias. It is simple, cheap, and requires minimal training, although it is limited to the treatment of more superficial and small lesions. We believe that this technique is suitable for office based setting. The advantage of using inexpensive and portable instruments will also be beneficial in developing counties where access to more expensive equipment is limited. Results are satisfactory but more patients are needed to validate the technique.

## INTRODUCTION

Telangiectasias are superficial cutaneous vessels of arteriolar, venule, or capillary origin.^1^ They appear as red, blue or purple linear marks in the skin. Arteriolar telangiectasias are smaller in diameter, with bright red appearance and do not protrude above the skin surface. Venule telangiectasias are wider, blue vessels with frequent protrusions on the skin surface. Capillary telangiectasias are often fine, red lesions, but can dilate and turn purple or blue over time due to venous backflow secondary to increased hydrostatic pressure.^2^ These lesions often cause noticeable aesthetic imperfections in the skin and patients frequently consult a specialist for treatment. 

**Fig. 1:**
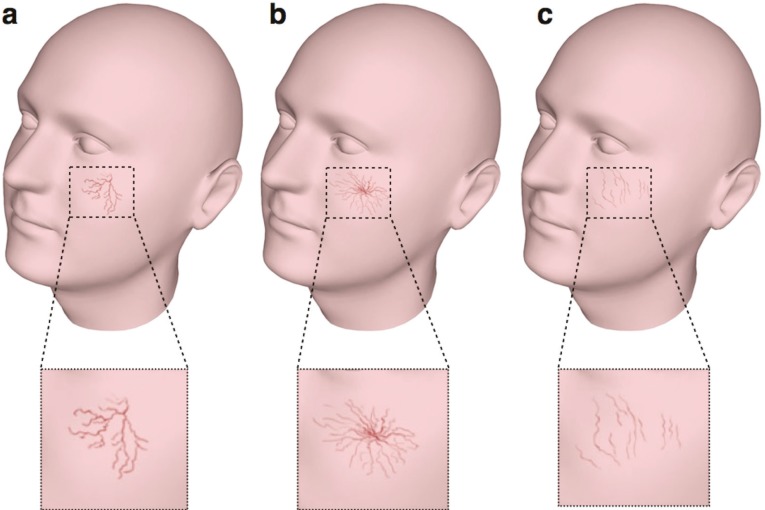
Types of telangiectasias. a) Arborizing (branch like), b) Spider (cartwheel shaped with a central point) and c) Simple, linear right

Based on the clinical appearance, telangiectasias are sub-classified into three types of arborizing, spider type and simple or linear (Figure 1). Red linear and arborizing telangiectasias are very common on the face (nose, mid cheeks and chin), while blue linear and arborizing telangiectasias are most commonly seen on the legs. Spider telangiectasias are common in childhood, and are of hereditary origin.^3^^,^^4^

Removal of all types of vascular lesions, including hemangiomas, port-wine stains, and telangiectasias, is performed using lasers, electrosurgery, sclerotherapy or surgical removal.^5^ Each technique has distinct advantages and limitations. For example, laser treatments, which involve *KTP, pulse dye, Alexandrite, Diode, ND-YAG *or* IPL* technology penetrate to the skin in different wavelengths (depending on the technology) in order to break the oxyhemoglobin and damage the vessel wall.^5^

However, they require extensive training and they can cause skin dyschromias. They are also expensive and need regular maintenance, thus increasing the cost of the treatment. Electrosurgery, a procedure by which a small cautery tip is applied on the skin along the length of the telangiectasia or at the root of the vessel, allowing electric current to coagulate the lesion. It is usually used in monopolar mode, it is simple and does not require extensive training, but it is contraindicated in patients with pacemakers. 

Sclerotherapy, a technique in which a sclerosing agent is injected inside the vessel, on the other hand, is effective for large vessels, but not a good option for small vessels. It removes the entire telangiectatic vessel but results vary significantly depending on the surgeon’s abilities and experience.^5^ This study presents a simple, safe, and cost-effective technique for the removal of facial telangiectasias. This technique requires minimal training and provides comparable results to the most common techniques used today. Further, it is associated with minimal, and transient complications.

## MATERIALS AND METHODS

**Fig. 2:**
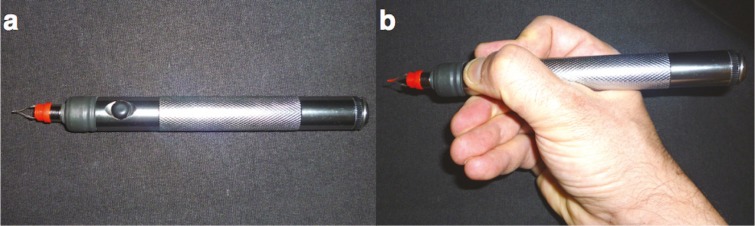
Hand cautery used for the treatment of the facial telangiectasias

This is a prospective non-randomized clinical study. Informed consent was obtained from all patients. Patient’s age ranged from 27 to 56 years old (Table 1). Twenty-five patients were treated by hand cautery (*Medicell Inc.*) (Figure 2) between 2009 and 2013 by a same plastic surgeon. Standardized pre- and post treatment photographs were taken. The area of the face intended for treatment was prepped with 70% alcohol. 

**Table 1:** Patients with facial telangiectasias

**Patient**	**Gender**	**Age**	**Size of the lesions (1-3)** *	**Treatments**
1	Female	46	3	3
2	Female	54	1	1
3	Female	28	2	2
4	Female	36	3	3
5	Female	39	1	2
6	Female	45	3	3 White scar formation
7	Female	56	1	1
8	Female	29	1	1
9	Female	38	1	1
10	Male	51	3	3White scar formation
11	Female	38	2	2
12	Female	44	1	1
13	Female	28	1	1
14	Female	41	3	3
15	Female	54	2	2
16	Female	56	1	1
17	Female	46	2	2
18	Female	47	3	2
19	Female	33	1	1
20	Female	38	2	1
21	Female	50	2	1
22	Female	28	3	2
23	Female	34	1	2
24	Female	37	3	2
25	Female	49	2	2

* Size of the lesion: 1 for facial telangiectasias around the nose, 2 for facial telangiectasias around the nose and the cheek and, 3 for facial telangiectasias around the nose, the cheek and the chin

Hand cautery was applied to each telangiectatic vessel using a 30G tip, inducing temporal heat elevation to ~800°C for milliseconds using low current energy conversion (1-2A). Entire vessel cauterization was performed by repeated applications of the tip over the entire length of the vessel. The procedure did not require local anesthesia and it was well tolerated by the patients. In most of the cases a single session was enough to remove the telangiectatic vessel, however, in some occasions, 2 or 3 consecutive sessions were necessary (each 3 weeks apart). Immediately after the treatment, an ice patch was used to cool the area, and topical antibiotic/cortisone cream *(Fucicort, Leo Pharmaceuticals) *was applied over the skin for 1-3 days to minimize bruising and erythema.

## RESULTS

**Fig. 3:**
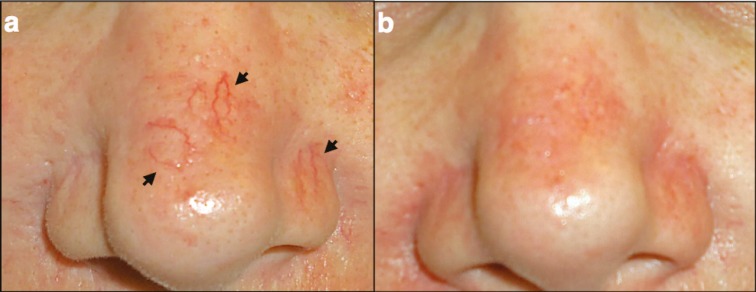
Patient with nose telangiectasias. a) Photos of a patent with lineal nose telangiectasia (black arrows); b) Photo of the same patient immediately after the treatment with hand cautery

**Fig. 4:**
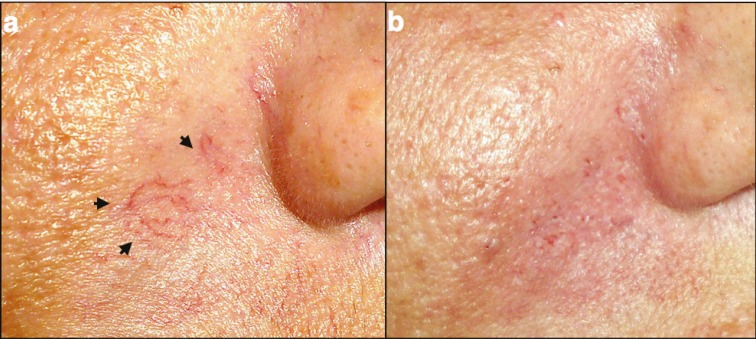
Patient with facial telangiectasias. a) Photos of a patent with arborizing facial telangiectasia (black arrows); b) Photo of the same patient immediately after the treatment with hand cautery

**Fig. 5:**
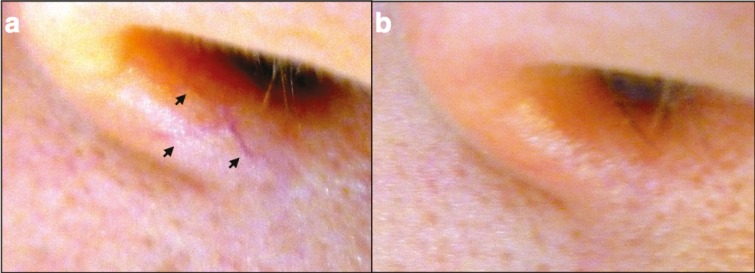
Patient with nose telangiectasias. a) Photos of a patent with spider nose telangiectasia (black arrows); b) Photo of the same patient immediately after the treatment with hand cautery

Twenty two out of 25 patients (88%) achieved dramatically improved in the appearance of facial telangiectasias, while 2 patients (8%) showed moderate improvement (Figure 3-5). A single session of hand cautery treatment completely removed the facial telangiectasias in 5 (20%) patients, while a repeated session after 3 weeks was required for 10 patients (40%). 

Five patients (20%) required 3 consecutive sessions, each separated by 3 weeks, in order to diminish the lesions. One patient (4%) showed no improvement after 3 treatments, likely due to deeper vessels location in the skin. The majority of the 25 patients (88%) exhibited minor complications, such as immediate post-operative edema, which resolved within 2-3 days using comfort measures such as massage and ice treatment. A white scar formation was visible in 2 patients (8%) but it become almost inconspicuous after 3 months with the application of a bleaching cream (Table 1). 

## DISCUSSION

Red linear and arborizing telangiectasias are very common on the face, especially the nose, mid cheeks and the chin. Several different treatment modalities have been proposed with each one having distinct therapeutic advantages and disadvantages. For example, sclerotherapy performed by injecting sclerosing agent into the vessel may cause endothelial damage, thrombosis, vessel wall necrosis and subsequent fibrosis. Even though it is a very efficient method for vessels greater than 0.4 mm in diameter,^4^^,^^6^ it has poor outcomes in vessels smaller than 0.2 mm in diameter and of arteriolar origin. In addition, injecting the sclerosing agent into the wrong vessel may cause overlying skin necrosis, and may even cause visual impairment due to infarction of the ophthalmic artery when the sclerosing agent is injected into periocular telangiectasias.^7^^-^^9^

Surgical removal has been anecdotally proposed for the treatment of facial telangiectasias, usually to the periorbital vessels. This technique requires a 2 mm incision under local anesthesia using a phlebectomy hook and it is considered technically challenging to perform with significant variability in results based on the surgeon’s experience. Electrosurgery with radiofrequency has shown efficacy in treating facial telangiectasias.^10^

The micro-coagulation-surgery utilizes a small cautery tip, similar to that used for hair epilation.^11^ Coagulation is performed by applying the tip on the skin along the length of the telangiectasia or at the root of the vessel, allowing electric current to coagulate the lesion. However, electric spreading over the skin may cause permanent hyperpigmentation or hypopigmentation in darker skins. The technique is also strictly prohibited in patients with pacemakers or implantable cardio-defibrillators.^11^

Bipolar coagulation,^12^ with or without topical anesthesia,^13^ can effectively remove very small facial vessels. Even though the technique is simple and effective, the equipment is expensive and difficult to obtain. Lasers can be used to remove facial telangiectasias through selective photothermolysis.^1^^,^^14^ Energy transferred to the oxyhemoglobin causes vessel wall damage. Many different laser wavelengths can be employed to perform this task, however each is associated with different complication. 532-595 nm (KTP, and pulse dye) lasers are contraindicated in darker skins due to the risk of causing scar.^15^^,^^16^

Alexandrite laser at 755nm has poor penetration into the dermis but it is less safe and less effective in dark-skinned patients.^17^ Diode lasers at 810-980nm are safe in treating facial telangiectasias, but require multiple treatments to achieve satisfactory results.^18^^-^^20^ IPL (Intensive Pulsed Light) technology incorporates a range of wavelengths with different skin penetration from superficial to deeper vessels.^18^ It is more suitable for the smaller vessels and requires experience by the Surgeon. Post-treatment, sun exposure is prohibited due to the risk of developing skin blisters and post-inflammatory hyperpigmentation, especially in darker skin types using short wavelengths.^21^

ND-YAG laser at 1064 nm has good tissue penetration and can treat vessels up to 3-4mm in diameter. It is suitable for skin type’s I-VI with or without a tan^22^^,^^23^ and for the treatment of lower extremity vessels.^24^ However, treatment of facial telangiectasias should always be performed in conjunction with a cooling system due to the high risk of developing skin blisters and scars.^25^

Our technique using hand cautery was proven safe and effective for the treatment of superficial and small (<1 mm in diameter) facial telangiectasias. A single fine electrode, driven by 1-2 A, generated heat (~ 800°C) that was delivered directly to the vessel causing cauterization. This technique may cause damage to the surrounding tissue as a result of dehydration and cellular fluids evaporation, however, this can be avoided by reducing the exposure time of the tip to the vessel and by providing cooling during or immediately after the treatment. Optimal results may require 3-4 sessions with 3 weeks intervals for efficient removal of facial telangiectasias. 

Hand cautery is a very safe and effective tool for the treatment of facial telangiectasias. It is simple, cheap, and requires minimal training although it is limited to the treatment of more superficial and small lesions. We believe that this technique is suitable for office based setting. The advantage of using inexpensive and portable instruments will also be beneficial in developing counties where access to more expensive equipment is limited. Results are satisfactory but more patients are needed to validate the technique. 

## Note

Please cite this paper as:

Liapakis IE, Englander M, Sinani R, Paschalis EI. Management of Facial Telangiectasias with Hand Cautery. World J Plast Surg 2015;4(2):127-133.
